# Altered Cytokine‐Induced STAT3 and STAT5 Activation of Peripheral T Follicular Helper Cells Contributes to Vaccine‐Non‐Responsiveness in Aging and HIV

**DOI:** 10.1111/acel.70438

**Published:** 2026-03-09

**Authors:** Sheldon Davis, Jonah Kupritz, Prabhsimran Singh, Savita Pahwa, Suresh Pallikkuth

**Affiliations:** ^1^ Department of Microbiology and Immunology University of Miami Miller School of Medicine Florida USA

**Keywords:** aging and HIV, Aging and humoral immunity, HIV and Immunity, IL‐2 and IL‐21, influenza vaccines, STAT5, Aging and Immunity, T follicular helper cells and STAT5

## Abstract

Previous studies from our lab identified functional defects in antigen‐specific peripheral T follicular helper cells (pTfh), characterized by low IL‐21 and high IL‐2 production, contributing to non‐responsiveness to the influenza vaccine in both aging and HIV. This study investigated how IL‐21‐induced STAT3 and IL‐2‐induced STAT5 activation in pTfh cells affects vaccine responses in aging people with HIV (PWH) and those without HIV (PWoH). Ninety participants, including young (Y, ≤ 40 years) and old (O, ≥ 65 years) PWoH (YPWoH, *n* = 23; OPWoH, *n* = 25) and virally suppressed PWH (YPWH, *n* = 19; OPWH, *n* = 23), received the seasonal quadrivalent influenza vaccine. Samples were collected at pre‐vaccination (day 0) and at days 14 and 28 post‐vaccination. Participants were classified as vaccine responders (VR) or non‐responders (VNR) based on serum antibody titers against vaccine antigens using the hemagglutination inhibition assay. Phosphoflow cytometry was performed on pre‐vaccination PBMCs stimulated with IL‐21 or IL‐2, and high‐dimensional analysis was performed using OMIQ software. Peripheral Tfh cells of young individuals showed greater IL‐21‐induced STAT3, reduced IL‐2‐induced STAT5 activity, and a reduced frequency of IL‐2R+ pTfh cells compared to older individuals. IL‐21‐induced STAT3 in naïve CD4+ T cells in young participants correlated with the frequency of pTfh cells. Among VNR, IL‐2‐induced STAT5 in pTfh cells inversely correlated with day 28 vaccine titers. Our findings emphasize the essential role of IL‐21 and IL‐2‐induced STAT signaling in orchestrating the immune response to vaccination. As individuals age, IL‐2‐induced STAT5 signaling in pTfh increases, potentially hindering Tfh cell differentiation and function, which may result in weaker vaccine responses.

## Graphical abstract

Chronic immune activation and inflammation associated with aging and HIV skew Tfh signaling toward enhanced IL‐2–STAT5 activation, reinforcing IL‐2R expression through a positive feedback loop and suppressing Tfh helper differentiation programs, thereby contributing to impaired vaccine responsiveness.

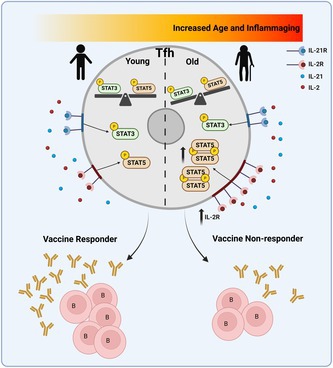

## 1 Introduction

With the success of antiretroviral therapy (ART), people with HIV (PWH) now have a life expectancy similar to that of people without HIV (PWoH) (Marcus et al. 2016; Trickey et al. 2023). However, PWH continue to experience chronic immune activation and inflammation, leading to a higher risk and earlier onset of developing non‐AIDS‐related comorbidities associated with immunosenescence (Deeks 2011; Deeks et al. 2012). Using an immunological age prediction approach, we previously demonstrated that young PWH experience accelerated immune aging of 5.6 years, while older PWH showimmune aging profiles similar to their age‐matched PWoH counterparts (de Armas, Pallikkuth, et al. 2021). Chronic immune activation and premature immunosenescence may impair the ability to mount protective antibody responses to infection or vaccination in both physiologically aged individuals and older individuals with HIV (Ferrucci and Fabbri 2018; Pereira et al. 2020; Sieg et al. 2021). Older adults are at higher risk for severe complications from vaccine‐preventable diseases, such as influenza (flu), and they account for > 70% of flu‐related hospitalizations (Centers for Disease Control and Prevention 2023; Naquin et al. 2024). We and others have reported poor serological response to flu vaccination in the context of aging and HIV infection (Dang et al. 2023; Herati et al. 2014; Hove‐Skovsgaard et al. 2020; Kaw et al. 2020; Pallikkuth et al. 2018, 2019, 2012; Rinaldi et al. 2017; Tortellini et al. 2023).

T‐follicular helper (Tfh) cells, a subset of helper CD4+ T cells characterized by high expression of the follicle homing receptor CXCR5, play a crucial role in regulating humoral immunity by supporting B cells in germinal centers (King et al. 2008). Tfh cells help antigen‐primed follicular B cells to undergo proliferation, immunoglobulin class switch recombination, affinity maturation, and differentiation into long‐lived plasma cells or memory B cells through the secretion of IL‐21 and co‐stimulatory molecule interactions (King et al. 2008). After leaving the lymph node, Tfh cells can enter the circulation as peripheral Tfh (pTfh) cells and continue to aid in B cell function (He et al. 2013). In the periphery, pTfh cells partially retain some of the phenotypic and functional characteristics of Tfh cells in the lymph node, providing a useful model to study lymphoid immune cell dynamics during infection and vaccination in the peripheral blood (Hill et al. 2021; Moysi et al. 2018; Pahwa 2019; Pallikkuth et al. 2019; Vella et al. 2019).

The maintenance and differentiation of Tfh cells from naïve CD4+ T cells are regulated by a network of transcription factors influenced by cytokine‐STAT signaling pathways (Ji et al. 2020). Once primed in the lymph node T cell zone, Tfh cell differentiation is driven by IL‐12 and autocrine IL‐21 signaling via a STAT3‐dependent pathway (Ma et al. 2009; Schmitt et al. 2014). This process leads to distinct phenotypic characterestics, including high expression of CXCR5, PD‐1, and ICOS (Eddahri et al. 2009; Vogelzang et al. 2008). IL‐21 acts through STAT3 to further increase IL‐21 production and promote the expression of Tfh associated transcription factors Ascl‐2, c‐Maf, and Bcl6 (Avery et al. 2010; Powell et al. 2019; Vogelzang et al. 2008). Conversely, IL‐2‐induced STAT5 is essential for the expression of the Tfh antagonist Blimp‐1, which antagonizes Tfh differentiation and B cell helper function by suppressing the CXCR5, c‐Maf, Bcl6, basic leucine zipper transcription factor ATF‐like transcription factor (Batf), and IL‐21 production (Johnston et al. 2009; Nurieva et al. 2012; Turner Jr. et al. 1994). Although IL‐2 and IL‐21 primarily signal through STAT5 and STAT3 pathways, respectively, these cytokines can activate both STAT proteins depending on cellular context, receptor expression levels, and the presence of other cytokines or environmental cues (Delespine‐Carmagnat et al. 2000; Johnston et al. 1995; Ng and Cantrell 1997; Spolski and Leonard 2008; Zeng et al. 2007).

We previously identified phenotypic and functional differences in antigen‐specific pTfh cells that are associated with non‐responsiveness to seasonal flu vaccination in older individuals and PWH (Pallikkuth et al. 2019). In vaccine non‐responders (VNR), pTfh cells showed reduced IL‐21 production and ICOS expression, along with increased IL‐2 production, exhibiting a pro‐inflammatory phenotype characterized by high IL‐17 and TNF production upon in vitro antigen stimulation (George et al. 2015; Pallikkuth et al. 2019). In contrast, vaccine responders (VR) demonstrated an expansion of both total and antigen‐specific pTfh cells at days 7 and 28 post‐vaccination with elevated IL‐21 and ICOS expression correlating strongly with serological antibody responses (Pallikkuth et al. 2019). Moreover, VNR showed high baseline immune activation and increased expression of the immune checkpoint marker PD‐1, which negatively correlated with serological responses post‐vaccination (Pallikkuth et al. 2019).

Given the critical role of pTfh cell function in vaccine‐induced antibody responses, we hypothesized that age and HIV status influence cytokine‐induced STAT5 and STAT3 signaling pathways in pTfh cells, potentially reducing vaccine responses. In this study, we combined high‐dimensional analysis of immunophenotyping and phosphoflow cytometry assays with detailed hemagglutination inhibition (HAI) antibody titer data following flu vaccination to assess how cytokine‐induced activation of STAT3 and STAT5 in pTfh cells affects immunocompetence in the context of aging and PWH with suppressed infection.

## 2 Results

### 2.1 Study Cohort Description and Validation of Vaccine Responder Classification

**FIGURE 1:**
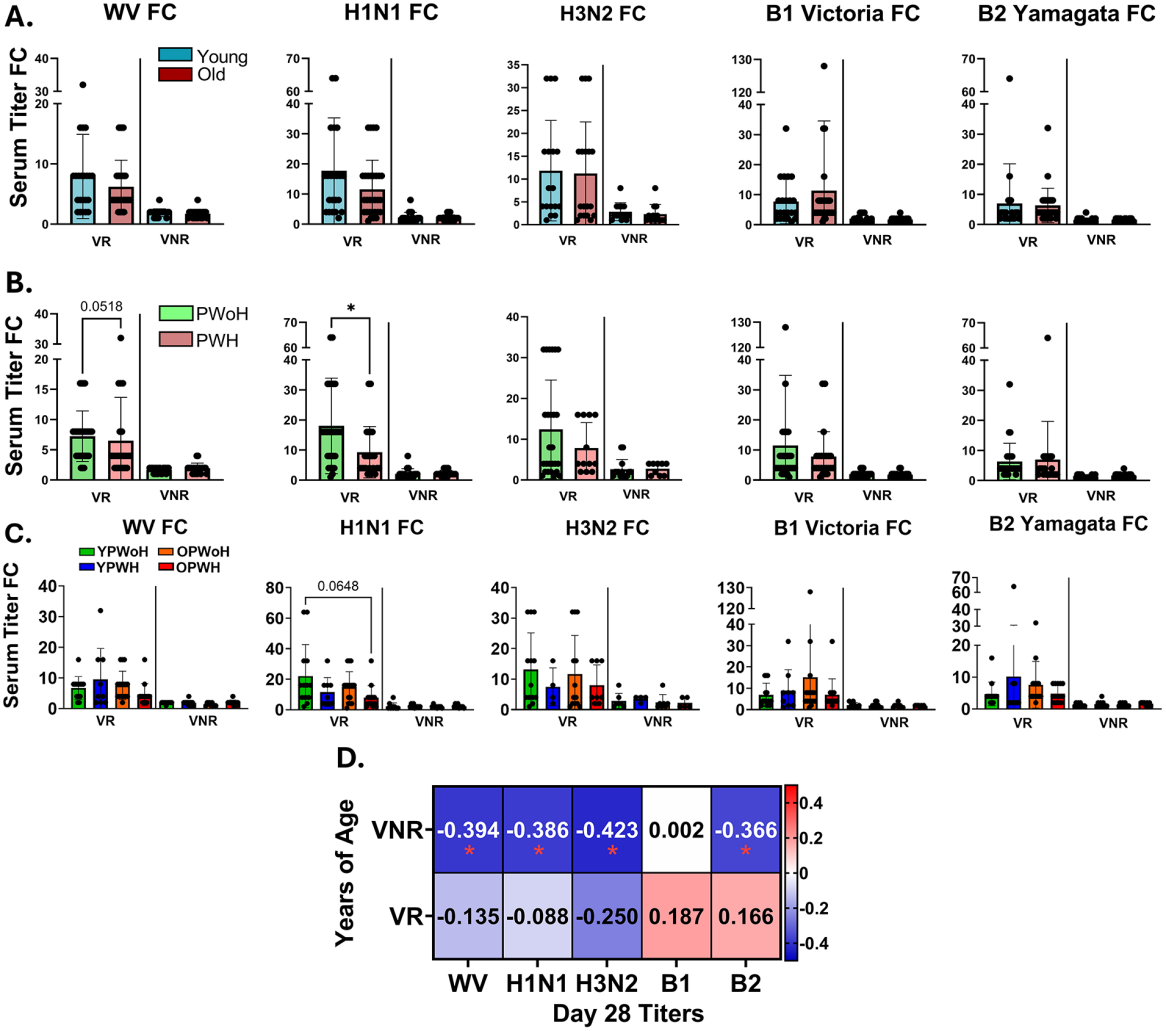
Negative associations between aging and vaccine response are most pronounced among vaccine non‐responders. Comparison of HAI titer fold‐change (FC) from 0 to 28 dpv between vaccine‐responder (VR) and vaccine non‐responders (VNR) separated by (A) age (young, blue, *n* = 42; old, red, *n* = 48) and (B) HIV status (PWH, pink, *n* = 42; PWoH, green, *n* = 48). *p* values are from unpaired, two‐tailed *t*‐tests. Comparison of (C) HAI titer FC within VR and VNR across each of the groups (YPWoH, *n* = 23; YPWH, *n* = 19; OPWoH, *n* = 25; OPWH, *n* = 23). *p* values are from Kruskal–Wallis with Dunn's multiple comparisons tests. (D) Spearman correlation between chronological age and antigen‐specific HAI titer at 28 dpv for VR and VNR. *p < 0.05; **p < 0.01; ***p < 0.001.

The present study included 90 participants: 52 VR (YPWoH, *n* = 13; YPWH, *n* = 9; OPWoH, *n* = 16; OPWH, *n* = 14) and 38 VNR (YPWoH, *n* = 10; YPWH, *n* = 10; OPWoH, *n* = 9; OPWH, *n* = 9). Mean age was similar between the VR and VNR groups (Table 1). Mean flu vaccine score, a cumulative measure of the immune response to vaccine antigens, was 8 for the VR group and 2 for the VNR group. Absolute CD4+ T cell count did not differ between the age groups, while YPWH and OPWH had significantly lower CD4/CD8 ratios than their age‐matched PWoH counterparts, consistent with prior reports on virally suppressed PWH (Novak et al. 2022). Mean absolute CD4^+^ T cell counts were > 500 cells/μL, and CD4/CD8 ratios were > 1, indicating long‐term viral suppression on ART. Serum HAI titers against individual flu antigens were measured at baseline (day 0; pre‐vaccination), and at 7, 14, 28 and 180 days post‐vaccination (dpv), while vaccine score calculations were based on the 0 to 28 dpv fold‐change (FC) response (Figure S1). Geometric mean HAI titers at 14 and 28 dpv did not differ between the YPWoH, YPWH, OPWoH, or OPWH groups, as expected given the preselection of participants based on their vaccine responses (Figure S2). In univariate analyses, HAI titer FC from 0 to 28 dpv was significantly higher in the VR group than in the VNR group, regardless of age or HIV status, except for A/H3N2, which was not included in the vaccine score calculations (Figure S3A–C). Among VR participants, older individuals showed a trend of lower A/H1N1 and whole vaccine (WV) responses (Figure 1A). Additionally, among VR participants, PWH had significantly lower A/H1N1 responses (*p* = 0.013) and a trend toward a lower WV response (*p* = 0.052) compared to PWoH (Figure 1B). Stratified analyses by age and HIV status revealed a trend toward higher A/H1N1 responses in YPWoH compared to OPWH among VR participants (*p* = 0.065, Figure 1C). Age‐response associations were more pronounced among VNR participants, with HAI titers at 28 dpv against WV, A/H1N1, A/H3N2, and B/Yamagata antigens showing consistent inverse correlations with chronological age (Figure 1D), suggesting that age‐related immune dysfunction may be amplified in individuals predisposed to poor vaccine responsiveness.

**TABLE 1:** Characteristics of study participants.

Population (90)	Young PWoH	Young PWH	Old PWoH	Old PWH	*p*
*N*	23	19	25	23	
Median Age in Years (Range)	30 (23–40)	29 (23–40)	64 (60–85)	63 (60–68)	—
Sex					
Female	57% (13)	32% (6)	28% (7)	39% (9)	0.22
Male	43% (10)	63% (12)	72% (18)	61% (14)	
Unknown	—	5% (1)	—	—	
Race					
White or Caucasian	22% (5)	37% (7)	32% (8)	35% (8)	0.71
Black or African American	65% (15)	53% (10)	64% (16)	61% (14)	0.83
Native American	—	5% (1)	—	4% (1)	—
Asian	4% (1)	—	—	—	—
Multiracial/Mixed	—	—	—	—	—
Other race	9% (2)	—	—	—	—
Unknow	—	5% (1)	4% (1)	—	—
Ethnicity					
Hispanic or Latino	35% (8)	37% (7)	20% (5)	39% (9)	0.48
Vaccine response					
Vaccine responders (VR)	57% (13)	47% (9)	64% (16)	61% (14)	0.72
Vaccine non‐responders (VNR)	43% (10)	53% (10)	36% (9)	39% (9)	
VR Vaccine Score Average ± SD	8.23 ± 3.6	8.00 ± 3.8	8.75 ± 3.7	7.00 ± 7.8	—
VNR Vaccine Score Average ± SD	2.00 ± 3.9	2.30 ± 3.5	1.89 ± 3.8	2.33 ± 2.8	—
CMV Serostatus					
Seropositive	39% (9)	63% (12)	48% (12)	48% (11)	0.48
HIV status – median (IQR)					
Baseline CD4 T cell count (cells/μL) Average ± SD	878 ± 327.5	785 ± 360.5	766 ± 332.6	763 ± 438.6	0.35
Baseline CD4/CD8 T cell count ratio (cells/μL) Average ± SD	1.9 ± 0.54	1.02 ± 0.55	2.6 ± 1.3	1.1 ± 0.55	< 0.0001
Duration on ART (Years) (Average/Median ± SD)	—	8.3/5 ± 9.8	—	18.3/16 ± 8.8	—

### 2.2 Peripheral Tfh Cell Frequency Is Associated With Vaccine Responsiveness

**FIGURE 2:**
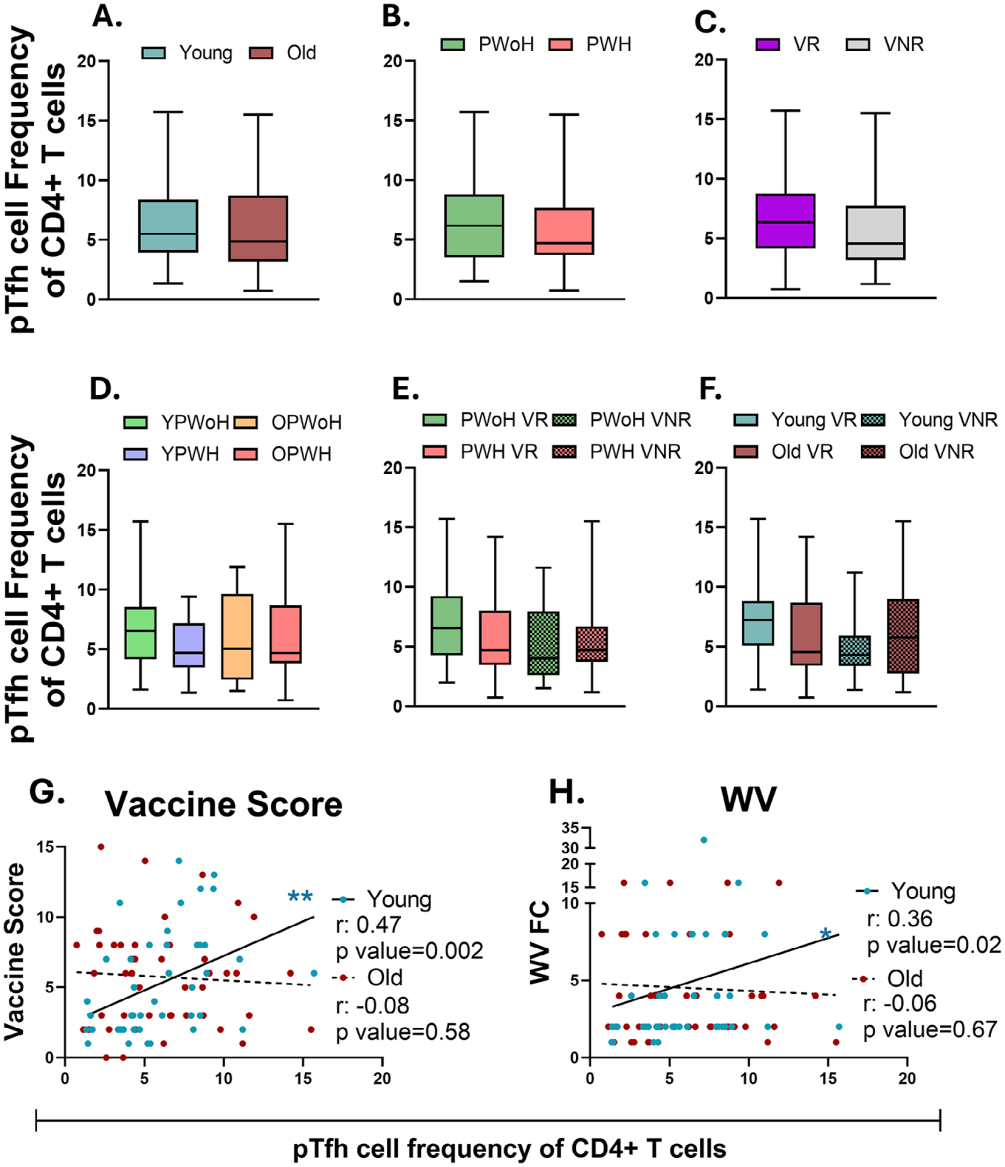
pTfh cell frequency in young individuals correlates with flu vaccine response. Comparisons of pTfh cell frequency in FOXP3‐ CD4+ T cells are based on (A) age (young, blue, *n* = 42 and old, red, *n* = 48), (B) HIV status (PWoH, green, *n* = 48 and PWH, pink, *n* = 42), and (C) vaccine‐responder group (VR, purple, *n* = 52; VNR, gray, *n* = 38). (D) Comparison of pTfh cell frequency between YPWoH (green, *n* = 23), YPWH (light purple, *n* = 19), OPWoH (orange, *n* = 25), and OPWH (red, *n* = 23). (E, F) Comparison of pTfh cell frequency between VR (solid bars) and VNR (checkered bars) separated by (E) HIV status and (F) age group. (G, H) Spearman correlations of pTfh cell frequency with (G) vaccine score or (H) whole vaccine fold‐change for the young (blue) and old (red) age groups. Mann–Whitney (A–C) or Dunn's multiple comparison tests (D–F). *p < 0.05; **p < 0.01; ***p < 0.001.

We next compared the frequency of pTfh cells across study groups and investigated their relationship with serological vaccine responses. Overall, pTfh frequency did not differ based on age group, HIV status, or vaccine response status, while YPWoH showed a trend of higher pTfh cell frequency compared to the other participant groups (Figure 2A–D). Mean pTfh frequency tended to be highest among PWoH and young participants categorized as VR (Figure 2E,F). Notably, among younger individuals, pTfh frequency positively correlated with vaccine score and WV HAI titer FC, whereas no such associations were found among older individuals (Figure 2G,H and Table S3). These findings align with prior studies by our group and others, indicating that a higher prevalence of pre‐vaccination Tfh cells is associated with enhanced vaccine‐induced antibody responses in YPWoH (George et al. 2015; Moysi et al. 2018; Pallikkuth et al. 2019).

### 2.3 Increased IL‐2‐Induced STAT5 and Reduced IL‐21‐Induced STAT3 During Aging With or Without HIV

**FIGURE 3:**
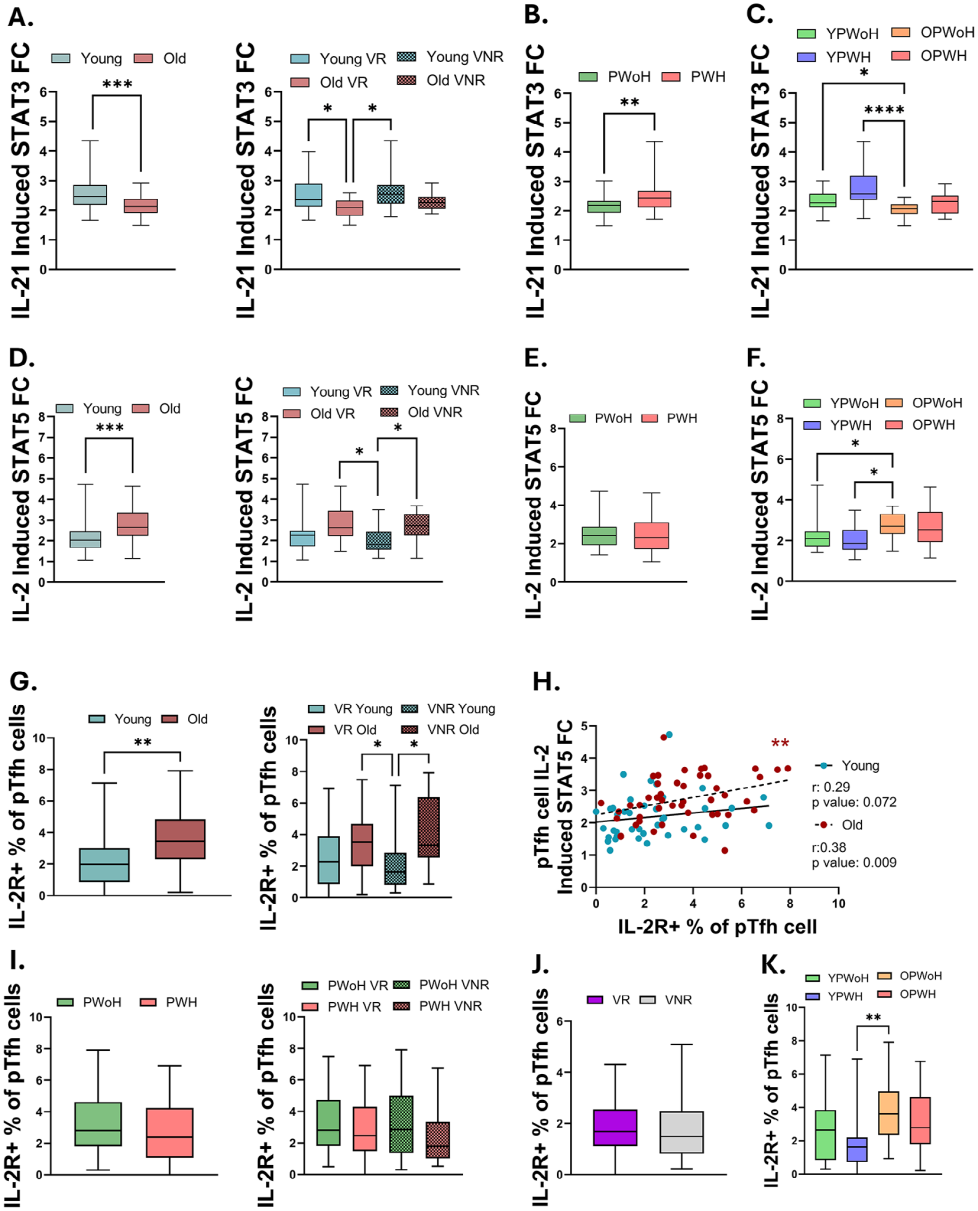
Older participants have higher pTfh IL‐2‐induced STAT5 and lower IL‐21‐induced STAT3 compared to younger participants. (A–C) Comparison of IL‐21‐induced STAT3 and (D–F) IL‐2‐induced STAT5 in pTfh cells by participant groups. Comparison of cytokine‐induced STAT based on (A, D) age (young, blue, *n* = 42; vs. old, red, *n* = 48), (B, E) HIV status (PWoH, green, *n* = 48 vs. PWH, pink, *n* = 42), and (C, F) study groups (YPWoH, (green, *n* = 23), YPWH (light purple, *n* = 19), OPWoH (orange, *n* = 25), and OPWH (red, *n* = 23)). (A, D, G, and I) Participants were classified as vaccine‐responders (VR, solid boxes) or vaccine non‐responders (VNR, checked boxes). (G–K) Frequency of IL‐2R+ pTfh cells compared between (G) age (young, blue, *n* = 40 and old, red, *n* = 46) and (I) HIV status groups (PWoH, green, *n* = 45 and PWH, pink, *n* = 41). (H) Spearman correlation analysis of IL‐2R+ pTfh cell frequency and pTfh cell IL‐2‐induced STAT5 between old and young participants. Comparison of IL‐2R+ pTfh cell frequency between (J) vaccine response groups (VR, purple, *n* = 49; VNR, gray, *n* = 37) and (K) study groups (YPWoH, green, *n* = 22; YPWH, light purple, *n* = 18; OPWoH, orange, *n* = 22; OPWH, red, *n* = 23).

Based on our previous observation that reduced IL‐21 and increased IL‐2 production by pTfh cells following in vitro antigen stimulation associated with poor influenza vaccine responses (Pallikkuth et al. 2019), we hypothesized that differential IL‐21‐induced STAT3 and IL‐2–induced STAT5 activation in pTfh cells contributes to impaired vaccine responsiveness in aging and HIV. We observed significantly higher IL‐21‐induced STAT3 activation in pTfh cells from younger individuals and VR compared to older individuals and VNR (Figure 3A). Across all participants, IL‐21‐induced STAT3 activation in pTfh cells negatively correlated with chronological age (Figure S4A). Unexpectedly, the pTfh IL‐21‐induced STAT3 response was higher among PWH than PWoH, primarily due to elevated responses in younger PWH, consistent with the overall age‐dependent pattern observed in the cohort (Figure 3B,C). In contrast, IL‐2‐induced STAT5 activation was higher among pTfh cells of older participants, regardless of vaccine response status (Figure 3D). Across all study participants, IL‐2‐induced STAT5 in pTfh cells positively correlated with age (Figure S4A), while no significant differences were observed in pTfh IL‐2‐induced STAT5 activation by HIV status (Figure 3E). When separated by age and HIV subgroups, YPWoH and YPWH showed lower IL‐2–induced STAT5 activation compared to OPWoH (Figure 3F).

Using a separate flow cytometry panel, we assessed the expression of the IL‐21 receptor (IL‐21R) and high‐affinity IL‐2 receptor (IL‐2Rα) in pTfh cells from the same participants. Older adults showed a significantly higher frequency of IL‐2Rα+ pTfh cells compared to younger participants (Figure 3G). Across all participants, the frequency of IL‐2Rα+ pTfh cells positively correlated with both pTfh IL‐2‐induced STAT5 activation and chronological age (Figure S4B). Age‐group stratified analyses revealed that IL‐2‐induced STAT5 activation positively correlated with the frequency of IL‐2Rα+ pTfh cells only among older participants, whereas no relationship was observed in younger participants (Figure 3H). No significant differences in the frequency of IL‐2Rα+ pTfh cells were found in relation to vaccine response or HIV status (Figure 3I,J). Analysis of combined age‐HIV subgroups revealed no differences based on HIV status within the young or old age groups, while age differences were primarily driven by increased IL‐2Rα in OPWoH compared to YPWoH (Figure 3K).

We next investigated the role of IL‐2‐induced STAT3 and IL‐21‐induced STAT5 activation in vaccine responses, since both cytokines can cross‐activate STAT3 and STAT5 to varying extents depending on receptor expression and cellular context. Older participants showed higher levels of IL‐2‐induced STAT3 activation in total CD4+ T and pTfh cells compared to younger participants (Figure S5A). Young VNR participants showed lower IL‐2‐induced STAT3 levels in pTfh and total CD4+ T cells compared to older VR and VNR participants (Figure S5A). In contrast, IL‐21‐induced STAT5 levels were higher in older participants, particularly among older VNR, across total CD4+ and pTfh cell compartments, regardless of vaccine response status (Figure S5C). HIV status was not associated with differences in either IL‐21‐induced STAT3 or IL‐21‐induced STAT5 levels (Figure S5B,D). Correlation analysis across all study participants revealed positive associations between IL‐21‐ and IL‐2‐induced STAT3 activation (*r* = 0.23, *p* = 0.03), IL‐2‐ and IL‐21‐induced STAT5 activation (*r* = 0.58, *p* < 0.0001) and IL‐2‐induced STAT5 and STAT3 activation (*r* = 0.40, *p* < 0.0001) in pTfh cells. Notably, the correlation between IL‐2‐induced STAT5 and STAT3 activation in pTfh cells was observed exclusively in older participants (Figure S5E). Conversely, correlations between IL‐21‐ and IL‐2‐induced STAT3 activation in pTfh cells were stronger in younger participants (Figure S5F). Together, these findings suggest that aging, but not HIV status, alters cytokine‐driven STAT signaling in pTfh cell subsets, potentially contributing to impaired adaptive immune responses. The increased IL‐2‐induced STAT3 activation in older participants may represent a compensatory mechanism for diminished IL‐21‐mediated STAT3 signaling with aging.

Finally, given the established relationship between aging related changes in pTfh cell frequency and vaccine responsiveness (George et al. 2015; Moysi et al. 2018; Pallikkuth et al. 2019), we investigated the association between pTfh frequency and cytokine‐induced STAT activation. Our findings revealed a positive correlation between pTfh frequency and the ratio of IL‐2‐induced STAT5 to IL‐2‐induced STAT3 among older individuals (*r* = 0.03, *p* = 0.041) and PWH (*r* = 0.38, *p* = 0.013). Among PWH classified as VNR, IL‐2‐induced STAT5 activation in pTfh cells showed a strong positive correlation with pTfh frequency (*r* = 0.71, *p* = 0.0006), a relationship not observed among VR.

**FIGURE 4:**
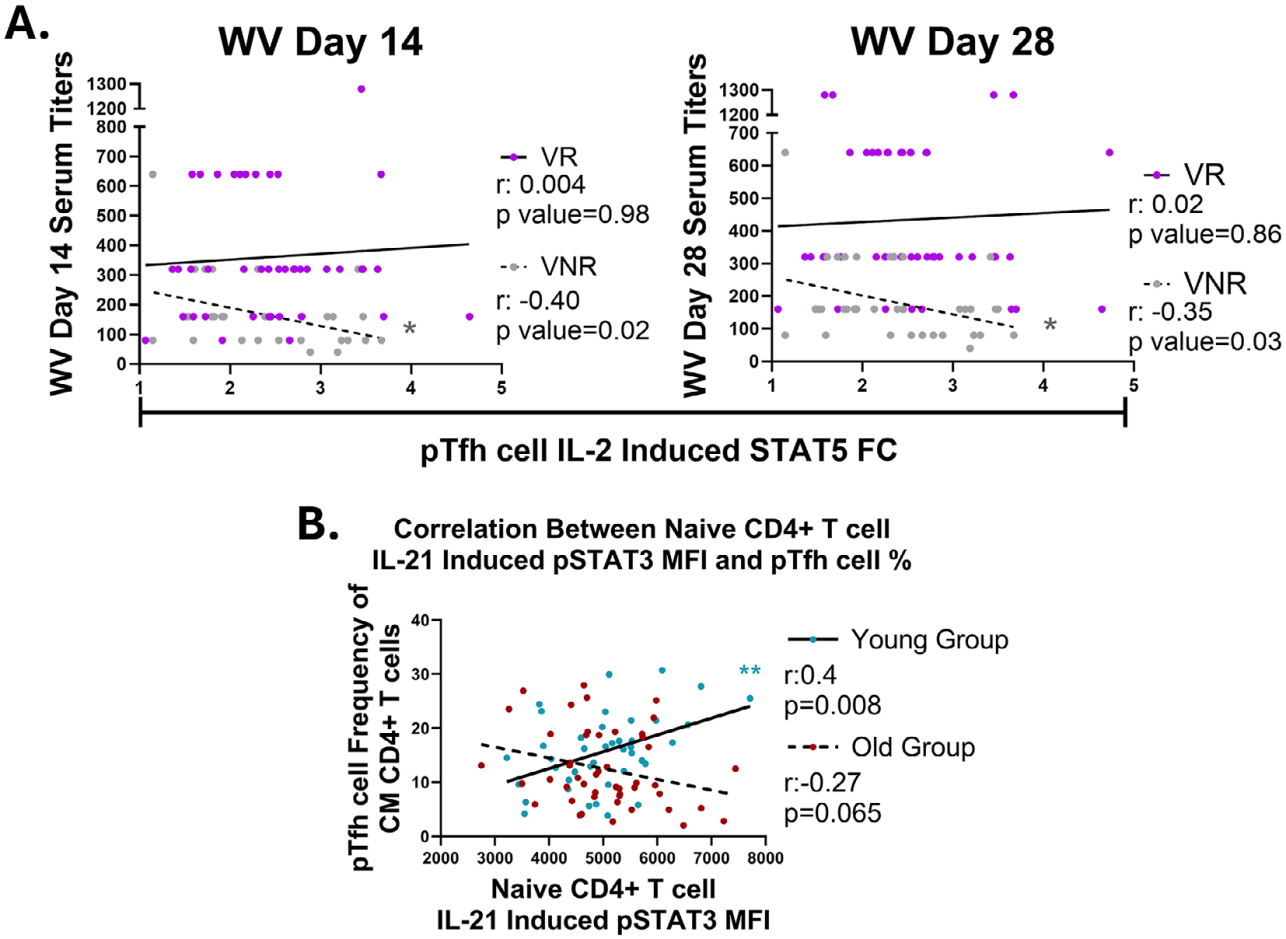
IL‐2‐induced STAT5 in pTfh is associated with diminished vaccine responses in non‐responders. (A) Spearman correlation between pTfh IL‐2‐induced STAT5 and HAI titers against WV in the vaccine non‐responder (VNR, gray, *n* = 38) and vaccine responder (VR, purple, *n* = 52) cohorts. (B) Spearman correlation between naïve CD4+ T cell IL‐21‐induced STAT3 and pTfh cell frequency in the central memory CD4+ T cell compartment for younger (blue, *n* = 42) and older (red, *n* = 48) groups. *p < 0.05; **p < 0.01; ***p < 0.001.

To assess a potential functional relationship between cytokine‐induced STAT activation and adaptive immunity, we correlated STAT activation with serological responses to the flu vaccine. In VNR, but not VR, IL‐2‐induced STAT5 activation in pTfh cells negatively correlated with WV HAI titer at both 14 and 28 dpv (Figure 4A). Within VNR, the inverse association between IL‐2‐induced STAT5 and WV HAI titers at 28 dpv was observed exclusively among PWoH (*r* = −0.66, *p* = 0.002).

### 2.4 Naïve CD4+ T Cells Support pTfh IL‐21‐Induced STAT3 Activation of Younger Participants

Across all participants, IL‐21‐induced STAT3 activation in pTfh cells directly correlated with IL‐21 induced STAT3 in the naïve CD4+ T cell compartment (*r* = 0.61, *p* < 0.0001). Additionally, IL‐21‐induced STAT3 in naïve CD4+ T cells positively correlated with the frequency of pTfh cells within the memory CD4+ T cell compartment (*r* = 0.40, *p* = 0.008) exclusively among young participants (Figure 4B). These findings suggest that IL‐21‐induced STAT3 activation in naïve CD4+ T cells may promote pTfh cell differentiation in younger individuals to enhance antigen responses, while age‐associated cell‐intrinsic immune alterations appear to limit this effect in older participants, with this association being attenuated with older age.

## 3 Discussion

Older adults and PWH face a heightened risk of severe flu‐related illness, while the flu vaccine, the primary strategy for prevention, elicits weaker antibody responses in these individuals (Crum‐Cianflone and Wallace 2014; Hou et al. 2024; Siberry et al. 2010). Extensive research has documented age‐associated impairment in vaccine responsiveness, which is further exacerbated in individuals aging with HIV (Crum‐Cianflone and Wallace 2014; George et al. 2015; Kernéis et al. 2014; Pallikkuth et al. 2018). Both aging and HIV contribute to the immune decline through overlapping mechanisms, including inflammaging, immunosenescence, and metabolic dysfunction (Lagathu et al. 2017; Pawelec et al. 1999). We previously reported immune dysfunction in virally suppressed PWH, characterized by increased immunosenescence, immune activation, and exhaustion compared to age‐matched PWoH, which correlated with diminished flu vaccine responses (de Armas et al. 2017; George et al. 2015; Parmigiani et al. 2013). Although strategies such as high‐dose or adjuvanted flu vaccines can partially mitigate the age‐related reduction in vaccine effectiveness, they do not address the underlying cell‐intrinsic immunological defects associated with aging and chronic HIV infection.

Peripheral Tfh cells play a central role in orchestrating humoral immune responses, and prior studies by our group and others have identified strong associations between pTfh frequency, function, and vaccine responsiveness (Hill et al. 2021; Kvistad et al. 2021; Pallikkuth et al. 2019, 2023; Yu et al. 2022). Notably, pTfh cells deficient in IL‐21 production, along with increased IL‐2 and TNF production and a shift toward an inflammatory Tfh1/Tfh17 phenotype upon antigen stimulation, contributed to vaccine non‐responsiveness (Pallikkuth et al. 2019). Mechanistically, optimal Tfh differentiation and function rely on the IL‐21‐STAT3 signaling axis, which drives Bcl6 expression and sustains the Tfh transcriptional program. In contrast, IL‐2‐induced STAT5 signaling antagonizes Tfh fate by upregulating Blimp‐1 expression, thereby promoting Th1 differentiation (Johnston et al. 2009; Kim et al. 2022; Nurieva et al. 2009). Based on our previous findings of an association between altered IL‐21 and IL‐2 pTfh responses, assessed using in vitro antigen‐induced marker assays, and impaired serological vaccine responsiveness (Pallikkuth et al. 2019), we designed this study to investigate whether age‐ and HIV‐associated alterations in cell‐intrinsic IL‐2‐ and IL‐21‐induced STAT3 and STAT5 activation in pTfh cells contribute to suboptimal immune responses to vaccination.

In line with our previous studies (George et al. 2015; Pallikkuth et al. 2018, 2019; Parmigiani et al. 2013), we observed a negative correlation between chronological age and vaccine responses, particularly among VNR. These findings suggest that age‐associated immune defects may reduce overall vaccine efficacy and predispose certain individuals to poor vaccine responsiveness, amplifying the impact of aging on humoral immune outcomes in this cohort. Supporting this observation, IL‐2‐induced STAT5 activation in pTfh cells inversely correlated with WV HAI titers in participants classified as VNR. Notably, older individuals exhibited increased IL‐2‐induced STAT5 activation in pTfh cells despite comparable pTfh frequencies across age, HIV status, and vaccine response status, indicating that cell intrinsic functional defects rather than quantitative deficiencies in pTfh cells impair Tfh mediated B‐cell help. Additionally, older participants displayed a higher frequency of IL‐2Rα+ pTfh cells, which correlated with increased IL‐2‐induced STAT5 activity. These findings suggest an age‐associated increase in IL‐2 responsiveness and STAT5 activity in pTfh cells that may compromise their functional capacity. When interpreting these results, it is important to distinguish between cytokine signaling sensitivity and maximal signaling capacity. The use of supraphysiologic concentrations of IL‐2 and IL‐21 (50 ng/mL) in this study allowed us to assess intrinsic STAT signaling capacity in pTfh cells, rather than differences driven by physiological cytokine availability or receptor engagement. Consistent with our findings, prior work demonstrated that naïve CD4+ T cells from older adults showed enrichment of STAT5 binding motifs within chromatin regions with age‐accelerated accessibility, increased IL‐2Rα transcription and protein expression, and elevated phosphorylated STAT5 and STAT5‐regulated transcription factors following TCR stimulation (Zhang et al. 2023).

Persistent immune activation and inflammaging, which are hallmarks of both aging and chronic HIV infection, likely drive sustained IL‐2‐induced STAT5 activity. Recurrent antigenic and inflammatory stimulation maintains elevated CD25 (IL‐2Rα) expression, reinforcing a positive IL‐2:IL‐2Rα signaling loop and prolonging STAT5 activation. This STAT5 dominant signaling environment may suppress IL‐21/STAT3 programs required for effective Tfh helper function. Studies have shown that Tfh cells from chronically aviremic PWH are dysfunctional due to an IL‐2–enriched microenvironment, characterized by reduced IL‐21 production and decreased CXCR5 and Bcl6 expression, alongside increased IL‐2–driven IFN‐γ and TNF‐α secretion (Cubas et al. 2015). Aviremic PWH also exhibit higher frequencies of IL‐2Rα‐expressing Tfh cells, which inversely correlate with Tfh cell frequency and total IgG production in vitro. Notably, exogenous IL‐2 supplementation in cocultures from elite controllers recapitulated this dysfunctional phenotype, leading to impaired B‐cell responses and enhanced pro‐inflammatory Tfh programs (Cubas et al. 2015). Consistent with this IL‐2‐dominant, STAT5‐skewed model of Tfh dysfunction, we previously showed that pTfh/B/APC cocultures stimulated with influenza antigen and treated with exogenous IL‐21 in combination with IL‐2 and TNF neutralization, exhibited enhanced IgG production in VNR participants, regardless of HIV status (Pallikkuth et al. 2019).

The negative regulatory mechanisms that typically attenuate IL‐2 signaling may be inadequate in aging and HIV infection to effectively downregulate IL‐2R expression between activation events. Following IL‐2 engagement, IL‐2Rβ and γc are degraded by lysosomes (Hémar et al. 1995), while IL‐2Rα is trafficked to early endosomes for recycling back to the cell surface (Su et al. 2015). Lysosomal activity supported by FOXO1 is reduced in naïve CD4+T cells from older adults, suppressing protein turnover (Jin et al. 2020). In PWH, intermittent IL‐2 therapy has been shown to induce a pool of IL‐2R^+^ naïve CD4^+^ T cells that persist for up to 5 years after treatment cessation (Sereti et al. 2002). Collectively, these findings suggest that chronic IL‐2 exposure may “lock” Tfh cells into a maladaptive state characterized by enhanced STAT5 signaling, impaired IL‐21 production, and defective B‐cell help. Regulation of the IL‐2:IL‐2R feedback loop is further influenced by metabolic stressors heightened in both aging and HIV infection. Additionally, while IL‐2 is a primary driver of STAT5 activation, elevated γc cytokines such as IL‐7 or IL‐15, which are known to increase in aging and chronic HIV infection (Gunst et al. 2023), may further sustain STAT5 signaling independent of IL‐2, reinforcing a STAT5‐dominant signaling environment in pTfh cells.

In line with recent studies (Bohacova et al. 2024), increased IL‐2‐induced STAT5 in the naïve CD4+ T cells from older participants is indicative of a signaling bias toward a pro‐inflammatory Th1 phenotype (Johnston et al. 2009; Jones et al. 2020; Nurieva et al. 2009). Beyond identifying pTfh STAT5 inhibition as a potential mechanism for enhancing antibody responses in aging, our research indicates that boosting IL‐21‐induced STAT3 activity in naïve CD4 T cell may significantly affect Tfh differentiation and function. In younger participants, IL‐21‐induced STAT3 activation in naïve CD4+ T cells correlated with a higher frequency of pTfh cells, highlighting the importance of the IL‐21‐STAT3 axis in maintaining the Tfh compartment. Consistent with their stronger vaccine responses, younger individuals showed higher IL‐21‐induced STAT3 and lower IL‐2‐induced STAT5 activity in pTfh cells compared to older participants. Collectively, these findings support a model where IL‐21‐driven STAT3 signaling in younger individuals promotes naïve CD4^+^ T cell differentiation toward the Tfh lineage and sustains the pTfh compartment, while enhanced IL‐2‐induced STAT5 signaling in naïve CD4^+^ T cells from older individuals skews CD4^+^ T cell differentiation toward inflammatory Th1‐like phenotypes, undermining effective Tfh responses.

Additional factors may influence STAT phosphorylation and vaccine responsiveness. Metabolic dysregulation associated with aging and HIV further reinforces STAT5 signaling dynamics. Quiescent memory T cells primarily rely on mitochondrial oxidative phosphorylation (OXPHOS) to maintain homeostasis (Quinn et al. 2019) and upregulate glycolysis and OXPHOS to rapidly produce ATP and intermediates needed for proliferation after activation (Shi et al. 2025). Aging is linked to higher basal and maximal OXPHOS capacity fueled by fatty acid oxidation, reduced glycolysis, increased mitochondrial content, and elevated reactive oxygen species production in memory CD4^+^ T cells, collectively promoting pro‐inflammatory cytokine production and inflammaging (Chen et al. 2022; Elyahu et al. 2019). Similarly, CD4+ T cells from virally suppressed PWH show an exhausted mitochondrial phenotype with increased mitochondrial mass and membrane potential (Akiso et al. 2023; Naidoo et al. 2024; Palmer et al. 2014), along with heightened glycolytic metabolism associated with increased proinflammatory cytokine production and highly glycolytic Th1/Th17 generation (Palmer et al. 2016). IL‐2 signaling activates the PI3K‐AKT‐mTORC1 pathway, enhancing mitochondrial function and glucose uptake, which shifts metabolism toward glycolysis and limits the generation of memory T and Tfh cell (Ray et al. 2015; Rollings et al. 2018). Previous studies have shown that glycolysis is suppressed by Bcl6, and IL‐2‐dependent glycolysis induction requires T‐bet, a Th1‐specific transcription factor (Oestreich et al. 2014). Aging and HIV may create an ATP‐rich cellular environment that favors JAK kinase activity, increasing STAT5 phosphorylation and promoting polarization away from the Tfh lineage.

Collectively, our data support a model in which age and HIV‐associated chronic immune activation, inflammation, and pTfh cell dysfunction are upstream factors that alter cytokine sensitivity and signaling balance, leading to increased IL‐2 and decreased IL‐21 responsiveness. This imbalance promotes sustained STAT5 activation at the expense of STAT3, reinforcing IL‐2R expression, suppressing IL‐21/STAT3‐dependent Tfh helper programs, impairing effective B cell help, promoting Th1‐like polarization, increasing proinflammatory cytokine production, and ultimately resulting in suboptimal vaccine outcomes. Our findings suggest that the combined assessment of IL‐2 induced STAT5 signaling, serum IL‐2 levels, and the frequency of IL‐2R+ Tfh cells may serve as potential predictive biomarkers of Tfh cell fitness and downstream serological outcomes. Further studies are needed to investigate additional mechanisms underlying STAT signaling defects in aging and HIV that may compromise overall immune competence. Additionally, future research should determine whether interventions such as IL‐21 augmentation at vaccination or modulation of STAT5 signaling in pTfh cells can reverse epigenetic and transcriptional alterations associated with aging, restore STAT signaling balance, mitigate inflammaging, and enhance Tfh cell function and vaccine responses in older adults and people with HIV.

Our study has several limitations that should be acknowledged. Although CD4+ T cell counts were comparable across aging and HIV groups, the baseline CD4+/CD8+ T cell ratio was higher in PWoH compared to PWH. This difference may indicate persistent immune reconstitution defects in PWH despite prolonged viral suppression. Infection from other pathogens, such as CMV, can also affect the CD4+/CD8+ T cell ratio and flu vaccine responses in aging (Manoharan et al. 2025); however, we did not find significant differences in CMV serostatus between the study groups (Table 1). While cytokines such as TGF‐β, IL‐12, and IL‐23 are known to direct naïve CD4^+^ T cells toward Tfh differentiation and lineage commitment within lymphoid tissues (Yoshitomi and Ueno 2021), these are beyond the scope of the current study. Our analyses focused on circulating peripheral Tfh cells, which represent mature, antigen‐experienced memory T cells already committed to the Tfh lineage. In addition, the number of participants in each VR and VNR subgroup was limited. Nevertheless, despite these constraints, we observed consistent findings across analyses. Finally, while we established a multicolor phospho‐flow cytometry approach to simultaneously assess STAT phosphorylation across multiple immune cell subsets, we were unable to comprehensively evaluate additional Tfh‐specific markers, including Bcl6, ICOS, and PD‐1. This limitation primarily stems from the rigorous fixation and permeabilization protocols, particularly those using high methanol concentrations, required for optimal STAT detection, which can compromise fluorochrome stability and antibody epitope integrity.

In conclusion, our findings demonstrate that the cytokine microenvironment and early cytokine‐induced STAT signaling at the site of the immune response critically shape CD4+ T helper differentiation toward the Tfh lineage, processes central to age‐associated immune dysfunction. These data support the hypothesis that dysregulation of the STAT3/STAT5 signaling axis plays a pivotal role in vaccine non‐responsiveness associated with aging. Further investigations are warranted to elucidate the mechanisms by which Tfh‐associated STAT3 and STAT5 signaling pathways contribute to impaired immunity in aging and aging PWH.

## 4 Materials and Methods

### 4.1 Study Participants and Sample Collection

Study participants included young (≤ 40 years, n=19) and older (≥ 65 years, n=23) virally suppressed PWH and PWoH (young *n* = 23, old *n* = 25) selected from an ongoing aging and HIV cohort (Table 1). Depending on the enrollment date, participants received the corresponding Fluzone Quadrivalent Influenza Vaccine (Table S1). All PWH were on ART with suppressed viremia; the mean ART duration was 8.3 years for young PWH and 18.3 years for older PWH (Table 1). CMV serostatus did not differ between groups (Table 1). The study was approved by the University of Miami Institutional Review Board (IRB protocol # 20200752) and written informed consent was obtained from all the participants. Peripheral blood samples were collected at pre‐vaccination (day 0) and 7, 14, 28, and 180 dpv. Peripheral blood mononuclear cells (PBMCs) were isolated by Ficoll–Hypaque density gradient centrifugation, and serum was aliquoted and stored at −80°C. PBMCs were cryopreserved in liquid nitrogen until analysis.

### 4.2 Hemagglutination Inhibition Assay and Vaccine Response Status Determination

The hemagglutination inhibition (HAI) antibody titer was measured for each vaccine antigen as previously described (de Armas, George, et al. 2021; George et al. 2018; Moysi et al. 2018; Pallikkuth et al. 2018, 2019, 2012). Seasonal flu vaccine antigens were generously provided by the Center for Biologics Evaluation & Research, Food & Drug Administration (CBER; FDA) as lyophilized vials. The flu vaccine score was calculated by summing the log2‐transformed fold change (d28/d0) responses to individual flu antigens that year: Σ (log2(d28/d0) A/H1 + log2(d28/d0) B/Vic + log2(d28/d0) B/Yam) (Abreu et al. 2020; Ghanta et al. 2024); A/H3N2 HAI responses were excluded from the vaccine score calculations because the A/H3N2 antigen for the 2022–2023 and 2023–2024 flu seasons did not agglutinate in the HAI assay, as noted by the CDC (Centers for Disease Control and Prevention 2022). Participants with a vaccine score ≥ 4 were classified as vaccine‐responders (VR) while those with a vaccine score ≤ 3 were considered vaccine non‐responders (VNR). In this study, we analyzed pre‐vaccination PBMCs from selected VR and VNR using phosphoflow techniques. To minimize potential confounding effects from high baseline antibody titers on study outcomes, individuals with baseline titers > 320 were excluded. This approach aimed to minimize potential distortions in the fold change (FC) response to individual antigens and to accurately determine VNR status, which could be affected by elevated pre‐vaccination titers.

### 4.3 Phosphoflow Cytometry and Immunophenotyping

We developed a 17‐color flow cytometry panel to study STAT phosphorylation in conjunction with cellular phenotype. Optimization and data collection was performed on a 5‐laser, 64 detected Aurora flow cytometer (Cytek Biosciences). Gain settings were calibrated daily using SpectroFlo QC Beads. Briefly, pre‐vaccination PBMCs were thawed, transferred to 2% fetal bovine serum (FBS) diluted in RPMI 1640 media, and incubated overnight at 37°C in a 5% CO_2_ humidified incubator. The following day, 10^6^ cells per well were plated in a 96 well V‐bottom plate and rested in the incubator for 1.5 hours. Media was discarded, and cells were resuspended in 50 μL of a pre‐fixation antibody master‐mix including LiveDead Blue, FcX block, CD4 BUV805, CD8 BV711, CD45 Alexa 532, CD20 BV570, CD19 cFlour BYG710, CCR4 APC‐Fire‐810, CD27 APC, CXCR3 APC‐Cy7, CCR6 BUV496, and CXCR5 BV480 (Table S2). Immediately after adding the pre‐fixation antibody master‐mix, cells were stimulated with 50 μL of IL‐2 (final concentration, 50 ng/mL), or IL‐21 (final concentration, 50 ng/mL) diluted in pre‐warmed 2% FBS medium and incubated for 15 min in 37°C water bath. Supraphysiologic concentrations of IL‐2 and IL‐21 (50 ng/mL) were used to assess maximal, cell‐intrinsic STAT3 and STAT5 signaling capacity independent of cytokine availability. To stop the reaction, 100 μL of BD Cytofix/Perm was added to fix the cells with incubation continued for an additional 10 min. Following fixation, cells were permeabilized by adding 200 μL of BD Perm III and incubating samples on ice for 30 min. Cells were rehydrated and washed twice with FACS wash buffer (0.5% FBS in PBS), followed by staining with the post‐permeabilization antibody master mix including pSTAT5 R718, pSTAT3 Alexa 488, pSTAT6 PE‐Cy7, CD45RO PE‐Cy5, CD3 BUV395, and FOXP3 PE (Table S2) for 30 min at room temperature. Finally, the cells were washed twice and resuspended in FACS wash prior to acquisition on the flow cytometer. Spectral unmixing and autofluorescence extraction were performed using Cytek SpectroFlo software. Flow cytometry data were analyzed using OMIQ software (Dotmatics) to exclude dead and doublet cells and to gate on Tfh and naïve CD4+ T cell populations (Figure S6). Cytokine‐induced fold change was determined by dividing the median fluorescence intensity (MFI) of cytokine‐induced STAT by the MFI of STAT in the media condition.

### 4.4 Peripheral Tfh Cell IL‐2R Expression

Surface expression of IL‐2R and IL‐21R on pTfh cells was analyzed by flow cytometry. Briefly, thawed PBMC were stained with LiveDead Blue followed by surface markers, CCR7 PerCP‐e710, CD27 eFluor 450, CD3 BUV395, CD45 BV570, CD45RA BV650, CD28 BV785, CD4 BUV805, CD25 (IL‐2R) BUV737, and IL‐21R BV421 at room temperature for 30 min. Cells were washed, fixed with 1% paraformaldehyde, and acquired on a Cytek Aurora Flow cytometer. Spectral unmixing and autofluorescence extraction were performed as described above. IL‐21R and IL‐2Rα expression were assessed in live, pTfh cells (LiveDead Blue‐ CD4+ CD45RA‐ CD27+ CXCR5+).

### 4.5 Statistical Analysis

Unpaired nonparametric tests, including the Mann–Whitney U test for two‐group comparisons and the Kruskal–Wallis test with Dunn's multiple comparisons for multigroup analyses, were used as needed. Correlation analyses were performed using Spearman's rank correlation coefficient. All statistical analyses and graphing were conducted using GraphPad Prism 10.

## Author Contributions

S.D.: Experimental design, performed experiments, data analysis, and manuscript draft preparation; J.K.: Data analysis and manuscript editing; P.S.: Performed experiments and data analysis; S.P.: Study design, funding acquisition, and manuscript review editing; Sur P. Study design, funding acquisition, data analysis, and manuscript review editing.

## Funding

This work was supported by a National Institute on Aging R01 grant to S. Pahwa and S. Pallikkuth (R01AG068110), and by the T32 Predoctoral Training in Translational Immunology Program, Department of Microbiology and Immunology at the University of Miami (T32AI162624), which provided support to S. Davis. The authors also acknowledge support from the Miami Center for AIDS Research Laboratory Core, funded by the National Institutes of Health (P30AI073961 to S. Pahwa).

## Conflicts of Interest

The authors declare no conflicts of interest.

## Supporting information


**Figure S1:** Schematic of participant sampling, HAI titer measurement, and group categorization for phosphoflow assay.
**Figure S2:** HAI titer values pre‐ and post‐vaccination of the participants selected for the phosphoflow assay. Kruskal–Wallis with Dunn's multiple comparison test compared the individual antigen titers responses between YPWoH, YPWH, OPWoH, and OPWH at a single timepoint. *p < 0.05; **p < 0.01; ***p < 0.001.
**Figure S3:** Comparison between VR and VNR HAI titer FC within aging and HIV status groups. Individual antigen HAI titer FC between VR (purple) and VNR (gray) within YPWoH, YPWH, OPWoH, and OPWH groups; (B) Young and old; (C) PWoH and PWH status groups. Univariate unpaired analysis (two groups) and Kruskal–Wallis (four groups) with Dunn's multiple comparison test compared between HAI titer FC between groups *p < 0.05; **p < 0.01; ***p < 0.001.
**Figure S4:** Association of pTfh cell IL‐2 and IL‐21 responsiveness with age and IL‐2Rα + pTfh cell frequency. (A) Spearman Correlation analysis between age and pTfh IL‐21‐induced STAT3 (green) and IL‐2‐induced STAT5 (red). (B) Spearman correlation between pTfh cell IL‐2‐induced STAT5 and the frequency of IL‐2Rα + pTfh cells and chronological age. Spearman correlational analysis. *p < 0.05; **p < 0.01; ***p < 0.001.
**Figure S5:** Peripheral Tfh cell IL‐2 and IL‐21 responsiveness in young and old participant groups. (A, B) pTfh IL‐2‐induced STAT3 and (C, D) IL‐21‐induced STAT5, separated by age group and HIV status. Participants were categorized as vaccine responders (VR, solid boxes) or vaccine non‐responders (VNR, checked boxes). Spearman correlation analysis of IL‐21‐induced STAT5 and IL‐2‐induced STAT3 versus (E) IL‐2‐indced STAT5 and (F) IL‐21‐induced STAT3, by age group. Comparisons were made using Mann–Whitney testing (two‐group), Dunn's testing (multi‐group), or Spearman correlational analysis. *p < 0.05; **p < 0.01; ***p < 0.001.
**Figure S6:** Phosphoflow panel, T and B cell gating strategy. Flow cytometry gating strategy illustrating the identification of lymphocyte within STAT activity was quantified. FoxP3+ Tregs and dead cells were excluded from analysis.

**Table S1:** Fluzone Quadrivalent Influenza Whole Vaccine composition for the 2021–2024 influenza seasons.
**Table S2:** Phosphoflow Cytometry Panel.
**Table S3:** Spearman Correlation between CM pTfh frequency and vaccine responses across the study groups.

## Data Availability

The data that support the findings of this study are available from the corresponding author upon reasonable request.
