# Supplementary material for: Altered Cytokine‐Induced STAT3 and STAT5 Activation of Peripheral T Follicular Helper Cells Contributes to Vaccine‐Non‐Responsiveness in Aging and HIV
Source: Aging Cell. 2026 Mar 9;25(3):e70438. doi: 10.1111/acel.70438 (PMC12970486; doi:10.1111/acel.70438)
Supplement: Supplementary file 1 — Figure S1: Schematic of participant sampling, HAI titer measurement, and group categorization for phosphoflow assay. Figure S2: HAI titer values pre‐ and post‐vaccination of the participants selected for the phosphoflow assay. Kruskal–Wallis with Dunn's multiple comparison test compared the individual antigen titers responses between YPWoH, YPWH, OPWoH, and OPWH at a single timepoint. *p < 0.05; **p < 0.01; ***p < 0.001. Figure S3: Comparison between VR and VNR HAI titer FC within aging and HIV status groups. Individual antigen HAI titer FC between VR (purple) and VNR (gray) within YPWoH, YPWH, OPWoH, and OPWH groups; (B) Young and old; (C) PWoH and PWH status groups. Univariate unpaired analysis (two groups) and Kruskal–Wallis (four groups) with Dunn's multiple comparison test compared between HAI titer FC between groups *p < 0.05; **p < 0.01; ***p < 0.001. Figure S4: Association of pTfh cell IL‐2 and IL‐21 responsiveness with age and IL‐2Rα + pTfh cell frequency. (A) Spearman Correlation analysis between age and pTfh IL‐21‐induced STAT3 (green) and IL‐2‐induced STAT5 (red). (B) Spearman correlation between pTfh cell IL‐2‐induced STAT5 and the frequency of IL‐2Rα + pTfh cells and chronological age. Spearman correlational analysis. *p < 0.05; **p < 0.01; ***p < 0.001. Figure S5: Peripheral Tfh cell IL‐2 and IL‐21 responsiveness in young and old participant groups. (A, B) pTfh IL‐2‐induced STAT3 and (C, D) IL‐21‐induced STAT5, separated by age group and HIV status. Participants were categorized as vaccine responders (VR, solid boxes) or vaccine non‐responders (VNR, checked boxes). Spearman correlation analysis of IL‐21‐induced STAT5 and IL‐2‐induced STAT3 versus (E) IL‐2‐indced STAT5 and (F) IL‐21‐induced STAT3, by age group. Comparisons were made using Mann–Whitney testing (two‐group), Dunn's testing (multi‐group), or Spearman correlational analysis. *p < 0.05; **p < 0.01; ***p < 0.001. Figure S6: Phosphoflow panel, T and B cell gating strategy. Flow cytometry gatin [file ACEL-25-e70438-s001.docx]

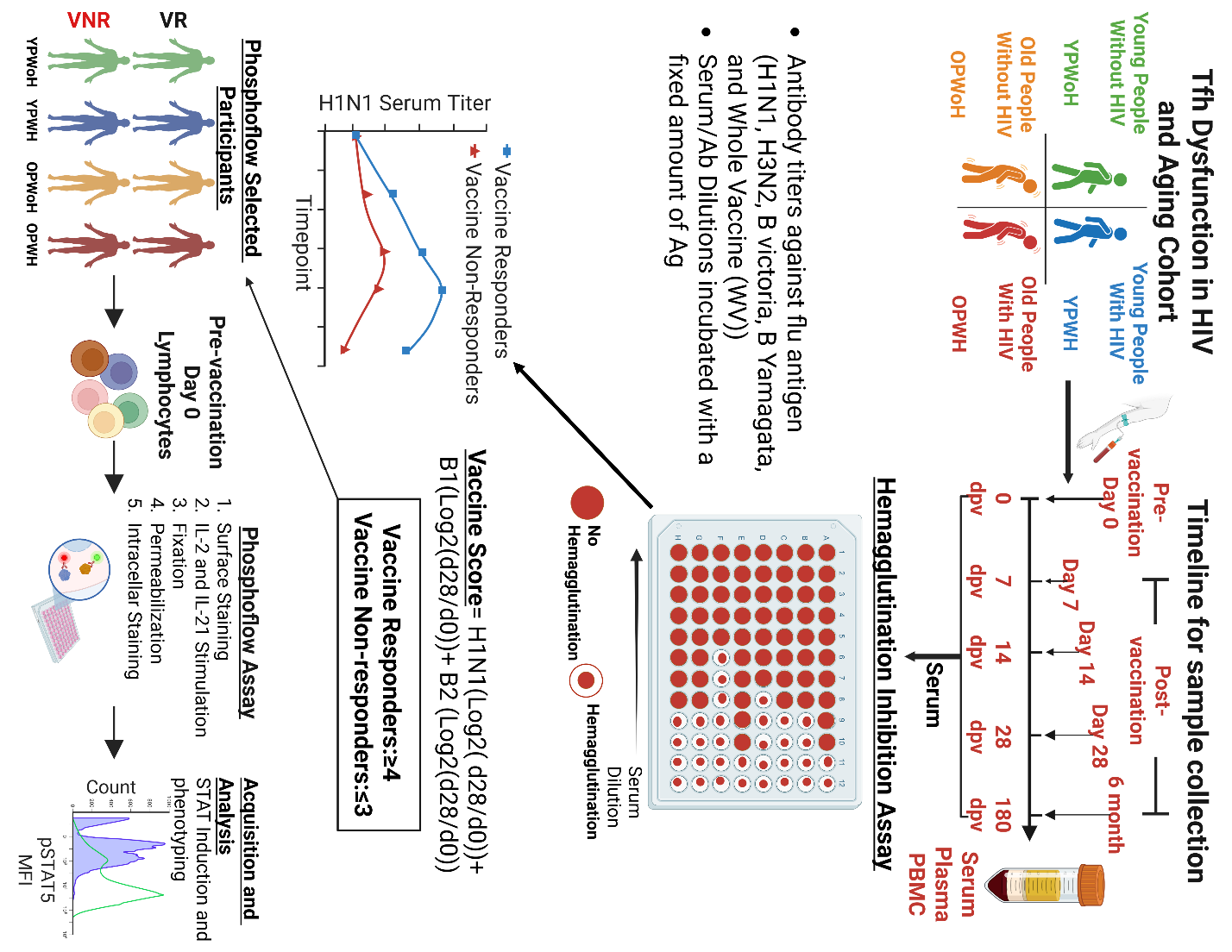


**Figure S1: Schematic of participant sampling, HAI titer measurement, and group categorization for phosphoflow assay**


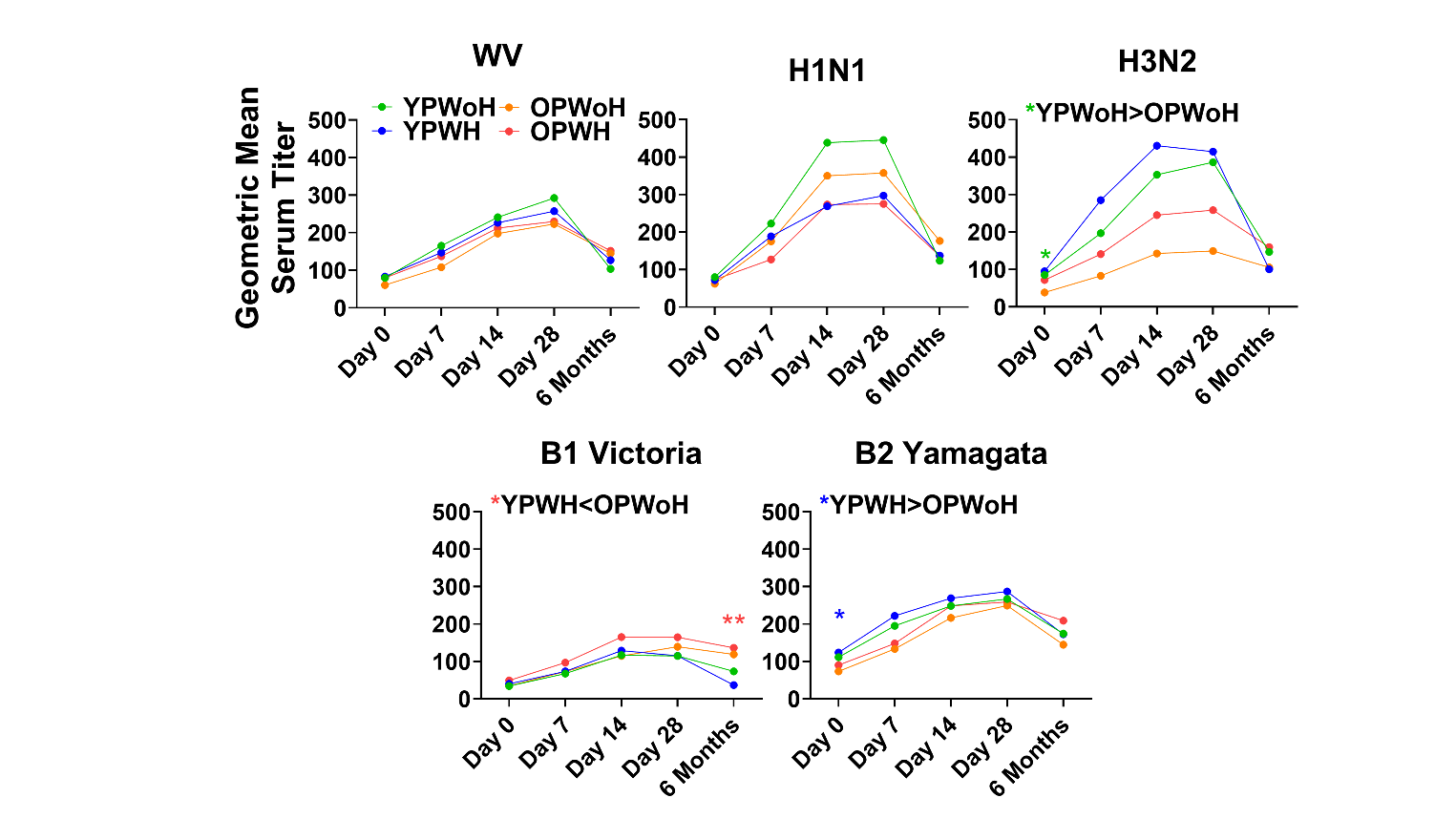


**Figure S2: HAI titer values pre- and post-vaccination of the participants selected for the phosphoflow assay.** Kruskal-Wallis with Dunn’s multiple comparison test compared the individual antigen titers responses between YPWoH, YPWH, OPWoH, and OPWH at a single timepoint. *P<0.05; **P<0.01; ***P<0.001.


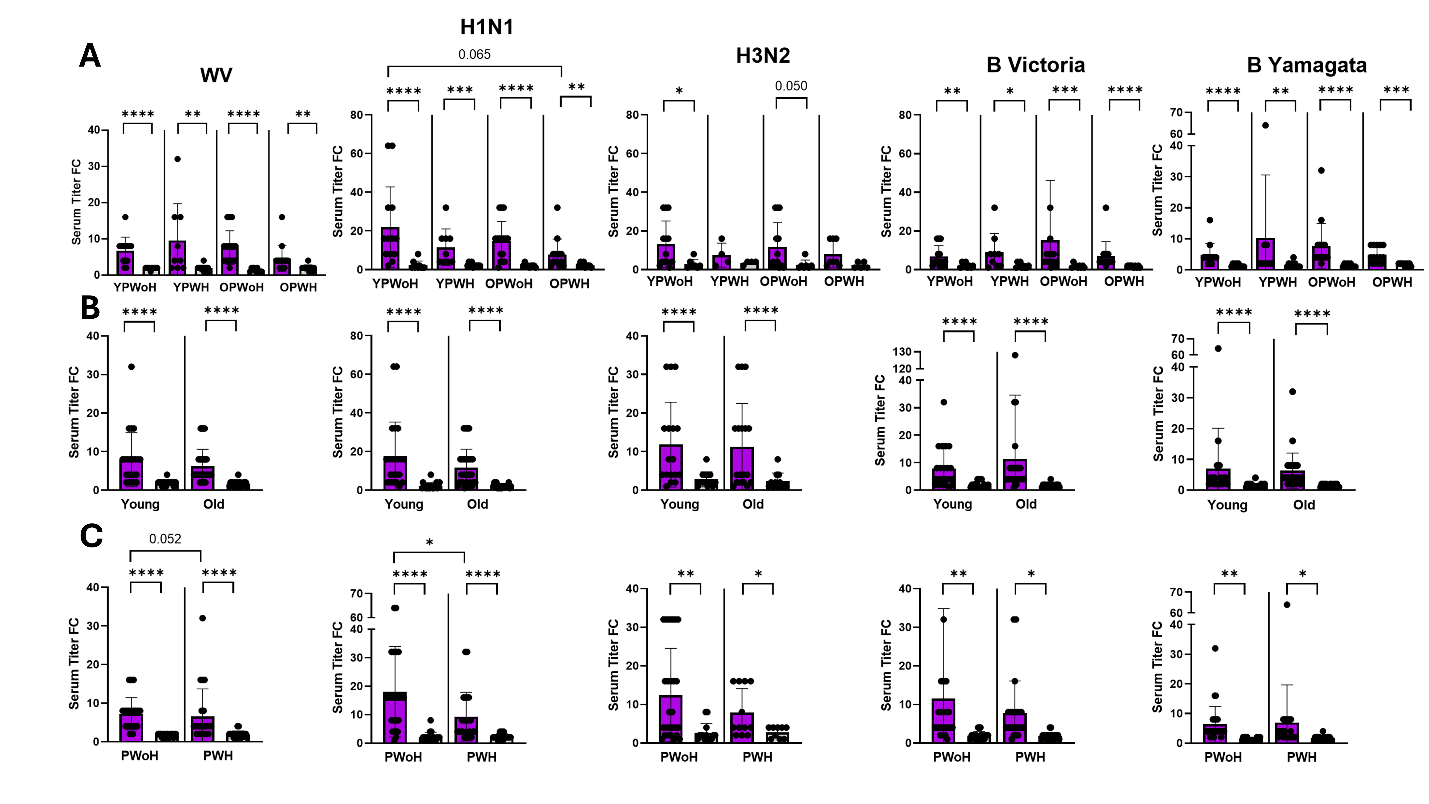


**Figure S3: Comparison between VR and VNR HAI titer FC within aging and HIV status groups.** Individual antigen HAI titer FC between VR (purple) and VNR (gray) within YPWoH, YPWH, OPWoH, and OPWH groups; (B) Young and old; (C) PWoH and PWH status groups. Univariate unpaired analysis (two groups) and Kruskal-Wallis (four groups) with Dunn’s multiple comparison test compared between HAI titer FC between groups *P<0.05; **P<0.01; ***P<0.001.


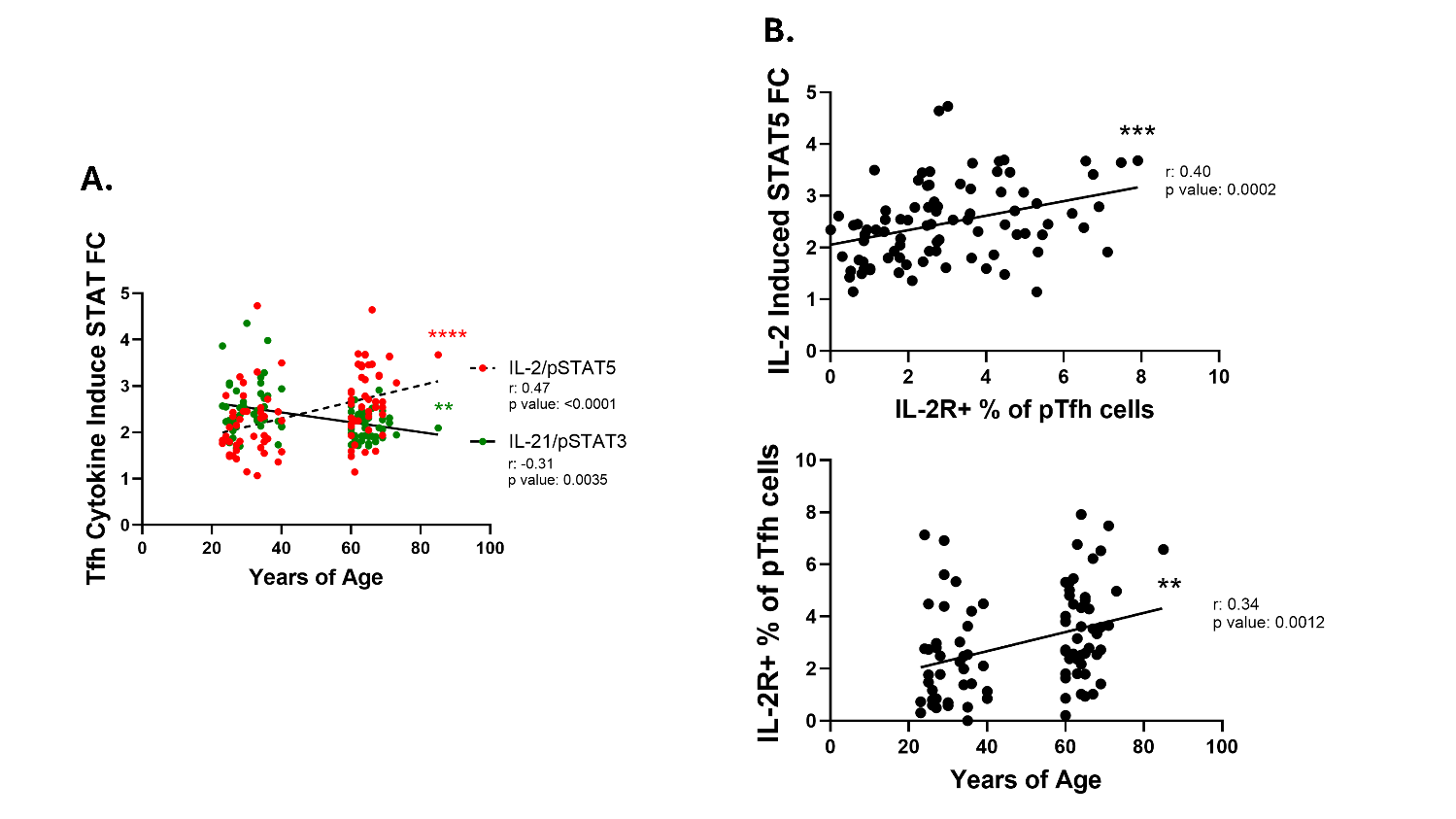


**Figure S4: Association of pTfh cell IL-2 and IL-21 responsiveness with age and IL-2Rα+ pTfh cell frequency.** (A) Spearman Correlation analysis between age and pTfh IL-21-induced STAT3 (green) and IL-2-induced STAT5 (red). (B) Spearman correlation between pTfh cell IL-2-induced STAT5 and the frequency of IL-2Rα+ pTfh cells and chronological age. Spearman correlational analysis. *P<0.05; **P<0.01; ***P<0.001.


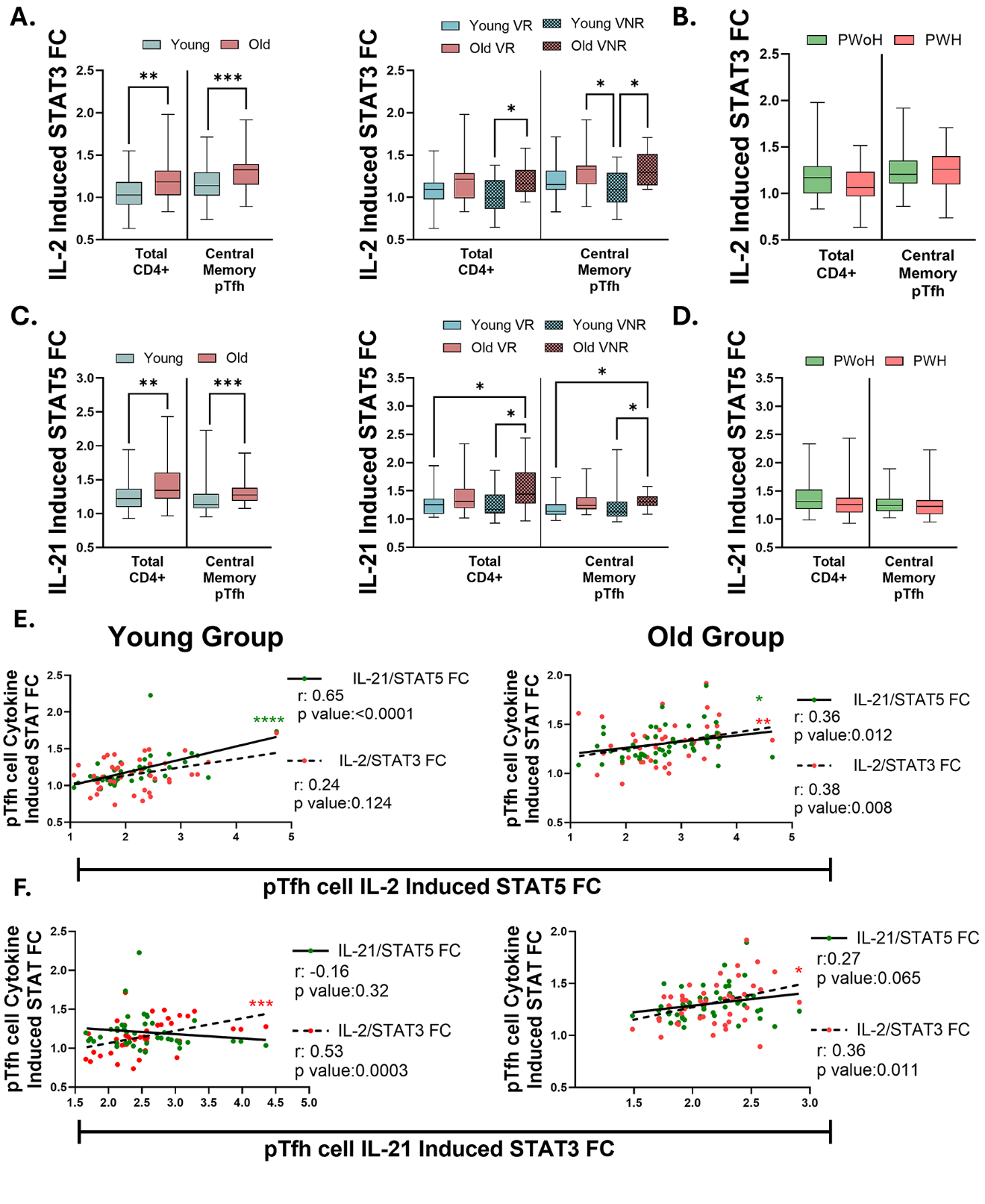


**Figure S5: Peripheral Tfh cell IL-2 and IL-21 responsiveness in young and old participant groups.** (A-B) pTfh IL-2-induced STAT3 and (C-D) IL-21-induced STAT5, separated by age group and HIV status. Participants were categorized as vaccine responders (VR, solid boxes) or vaccine non-responders (VNR, checked boxes). Spearman correlation analysis of IL-21-induced STAT5 and IL-2-induced STAT3 vs. (E) IL-2-indced STAT5 and (F) IL-21-induced STAT3, by age group. Comparisons were made using Mann-Whitney testing (two-group), Dunn’s testing (multi-group), or Spearman correlational analysis. *P<0.05; **P<0.01; ***P<0.001.


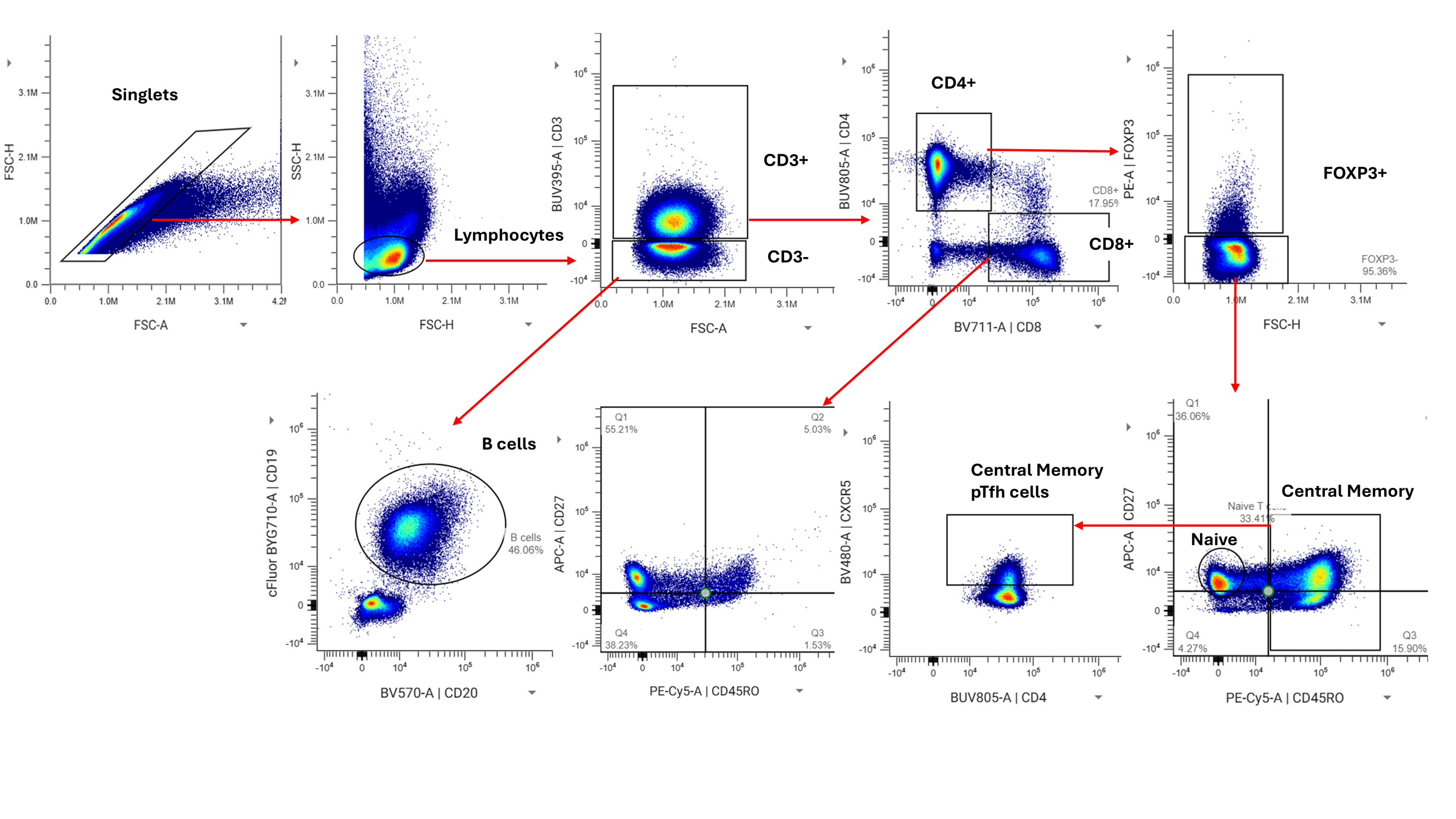
 **Figure S6: Phosphoflow panel, T and B cell gating strategy.** Flow cytometry gating strategy illustrating the identification of lymphocyte within STAT activity was quantified. FoxP3+ Tregs and dead cells were excluded from analysis.
