# Supplementary material for: Altered Cytokine‐Induced STAT3 and STAT5 Activation of Peripheral T Follicular Helper Cells Contributes to Vaccine‐Non‐Responsiveness in Aging and HIV
Source: Aging Cell. 2026 Mar 9;25(3):e70438. doi: 10.1111/acel.70438 (PMC12970486; doi:10.1111/acel.70438)
Supplement: Supplementary file 2 — Table S1: Fluzone Quadrivalent Influenza Whole Vaccine composition for the 2021–2024 influenza seasons. Table S2: Phosphoflow Cytometry Panel. Table S3: Spearman Correlation between CM pTfh frequency and vaccine responses across the study groups. [file ACEL-25-e70438-s002.docx]

**Supplementary Table S1: Fluzone Quadrivalent Influenza Whole Vaccine composition for the 2021-2024 influenza seasons**

| Season | | 2021-2022 | | 2022-2023 | | 2023-2024 | |
| --- | --- | --- | --- | --- | --- | --- | --- |
| A/H1N1 | A/Victoria/2570/2019 | | A/Victoria/2570/2019 | A/Victoria/4897/2022 | A/Victoria/2570/2019 | A/Victoria/2570/2019 | A/Victoria/4897/2022 |
| A/H3N2 | A/Tasmania/503/2020 | | A/Darwin/9/2021 | A/Darwin/9/2021 | A/Tasmania/503/2020 | A/Darwin/9/2021 | A/Darwin/9/2021 |
| B/Vic Victoria | B/Washington/02/2019 | | B/Michigan/01/2021 | B/Michigan/01/2021 | B/Washington/02/2019 | B/Michigan/01/2021 | B/Michigan/01/2021 |
| B/Yam Yamagata | B/Phuket/3073/2013 | | B/Phuket/3073/2013 | B/Phuket/3073/2013 | B/Phuket/3073/2013 | B/Phuket/3073/2013 | B/Phuket/3073/2013 |

**Supplementary Table S2: Phosphoflow Cytometry Panel**

| Detector | Specificity | Fluorochrome | Clone | Catalog # | Vendor |
| --- | --- | --- | --- | --- | --- |
| UV2 | CD3​ | BUV395​ | SK7 | 564117 | BD |
| UV6 | LiveDead Blue | ─ | - | L34955 | Invitrogen |
| UV16 | CD4 | BUV805 ​ | SK3 | 612887 | BD |
| V13 | CD8 | BV711 ​ | RPA-T8 | 301044 | Biolegend |
| B3 | CD45 | Alexa 532​ | HI30 | 2560265 | Life Techno-  logies |
| V8 | CD20​ | BV570 | 2H7 | 302330 | Biolegend |
| YG7 | CD19 | cFluor BYG710 | HIB19 | SKU R7-20010 | Cytek |
| R8 | CCR4 | APC-Fire-810​ | HIT2 | 359440 | Biolegend |
| R1 | CD27 | APC​ | M-T271 | 558664 | BD |
| YG5 | CD45RO​ | PE-Cy5​ | UCHL1 | 304208 | Biolegend |
| R7 | CXCR3 | APC-Cy7 | G025H7 | 353722 | Biolegend |
| UV11 | CCR6 | BUV496 | 11A9 | 612948 | BD |
| V5 | CXCR5 ​ | BV480 ​ | RF8B2 | 566142 | BD |
| R4 | pSTAT5​ | R718​ | 47/Stat5 | 566977 | BD |
| B2 | pSTAT3​ | Alexa 488​ | 13A3-1 | 651006 | Biolegend |
| YG9 | pSTAT6 | PE-Cy7​​ | A15137E | 686013 | Biolegend |
| YG1 | FOXP3 | PE​​ | 259D/C7 | 5323870 | BD |

**Supplementary Table S3: Spearman Correlation between CM pTfh frequency and vaccine responses across the study groups.**

|  | Young | | Old | | YPWoH | | YPWH | | OPWoH | | OPWH | | |
| --- | --- | --- | --- | --- | --- | --- | --- | --- | --- | --- | --- | --- | --- |
| HAI Titer | r | P | r | P | r | P | r | P | r | p | r | p |  |
| Vaccine Score | 0.474 | 0.001 | -0.08 | 0.588 | 0.49 | 0.017 | 0.37 | 0.112 | -0.01 | 0.968 | -0.29 | 0.177 |  |
| A/H1N1 FC | 0.414 | 0.007 | -0.001 | 0.990 | 0.45 | 0.033 | 0.30 | 0.220 | 0.02 | 0.915 | -0.09 | 0.668 |  |
| B/Yam FC | 0.393 | 0.012 | -0.05 | 0.740 | 0.51 | 0.014 | 0.26 | 0.280 | 0.03 | 0.869 | -0.23 | 0.298 |  |
| A/H1N1 Day 14 | 0.369 | 0.022 | -0.02 | 0.887 | 0.49 | 0.022 | 0.12 | 0.646 | 0.00 | 0.998 | -0.09 | 0.702 |  |
| B/Yam Day 14 | 0.465 | 0.003 | 0.005 | 0.971 | 0.47 | 0.029 | 0.55 | 0.028 | 0.05 | 0.804 | -0.20 | 0.379 |  |
| WV  Day 14 | 0.501 | 0.001 | -0.02 | 0.914 | 0.57 | 0.006 | 0.44 | 0.086 | 0.06 | 0.772 | -0.18 | 0.427 |  |
| A/H1N1 Day 28 | 0.374 | 0.014 | -0.05 | 0.758 | 0.51 | 0.011 | 0.06 | 0.802 | -0.03 | 0.891 | -0.12 | 0.573 |  |
| B/Vic Day 28 | 0.238 | 0.128 | -0.11 | 0.455 | 0.49 | 0.018 | 0.45 | 0.056 | -0.07 | 0.744 | -0.20 | 0.369 |  |
| WV  Day 28 | 0.485 | 0.001 | -0.001 | 0.996 | 0.51 | 0.012 | 0.41 | 0.079 | 0.09 | 0.659 | -0.18 | 0.400 |  |
